# Strategy for Leukemia Treatment Targeting SHP-1,2 and SHIP

**DOI:** 10.3389/fcell.2021.730400

**Published:** 2021-08-19

**Authors:** Fang Hao, Chen Wang, Christine Sholy, Min Cao, Xunlei Kang

**Affiliations:** Center for Precision Medicine, Department of Medicine, University of Missouri, Columbia, MO, United States

**Keywords:** SHP-1, SHP-2, SHIP, leukemia, AML, PTP inhibitor, signaling pathway, thrapeutic target

## Abstract

Protein tyrosine phosphatases (PTPs) are modulators of cellular functions such as differentiation, metabolism, migration, and survival. PTPs antagonize tyrosine kinases by removing phosphate moieties from molecular signaling residues, thus inhibiting signal transduction. Two PTPs, SHP-1 and SHP-2 (SH2 domain-containing phosphatases 1 and 2, respectively) and another inhibitory phosphatase, SH2 domain-containing inositol phosphatase (SHIP), are essential for cell function, which is reflected in the defective phenotype of mutant mice. Interestingly, SHP-1, SHP-2, and SHIP mutations are identified in many cases of human leukemia. However, the impact of these phosphatases and their mutations regarding the onset and progression of leukemia is controversial. The ambiguity of the role of these phosphatases imposes challenges on the development of targeting therapies for leukemia. This fundamental problem, confronted by the expanding investigational field of leukemia, will be addressed in this review, which will include a discussion of the molecular mechanisms of SHP-1, SHP-2, and SHIP in normal hematopoiesis and their role in leukemia. Clinical development of leukemic therapies achieved by targeting these phosphatases will be addressed as well.

## Introduction

**FIGURE 1:**
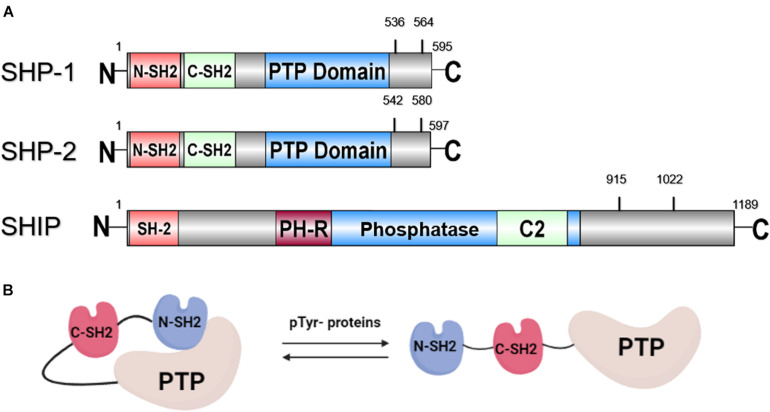
**(A)** Schematic representation of the structure of SHP-1, SHP-2, and SHIP with tyrosine phosphorylation sites. **(B)** Structure of SHP protein in auto-inhibited and activated conformation.

SH2 domain-containing phosphatase 1 (SHP-1), encoded by the *PTPN6* gene, is expressed mainly in hemopoietic cellular systems. SHP-1, with a molecular weight of 68 kDa, is made up of three domains: the N-terminal Src homology-2 (SH2) domain, the C-terminal SH2 domain, and the C-terminal catalytic Protein tyrosine phosphatase (PTP) domain (Figure 1A; Lorenz, 2009). Two tyrosine residues at the C-terminus of SHP-1 (Y536 and Y564) are phosphorylated by various stimuli. The phosphorylation of tyrosine residues modifies the function and activity of SHP-1 depending on the property of the stimulus. SHP-1 exists in an auto-inhibited conformation (Yang et al., 2003). The N-SH2 domain binds tightly to the PTP domain and blocks substrate access to the catalytic domain, thus keeping the enzyme in its inactive conformation. On the contrary, when the phospho-peptide is bound to the C-SH2 domain, the N-terminal SH2 domain is released from the PTP domain, which relieves this autoinhibition and catalytically activates the enzyme (Figure 1B). The “moth-eaten” mice demonstrated a deficiency of SHP-1 and were affected by many hemopoietic disorders, including autoimmune hyperactivation of macrophages, which that suggested a defect in negative regulation (Shultz et al., 1997; Tsui et al., 2006). The SHP-1 gene has two promoters: the distal promoter, which is only present in epithelial cells, and the proximal promoter, which is active in both epithelial and hemopoietic cells. Therefore, under normal conditions, expression levels of SHP-1 vary between epithelial and hematopoietic cells. This concept is supported by an aberrant level of SHP-1 in cancers (Tsui et al., 2006). In hematopoietic cancers, such as myeloma, promoter methylation inhibited SHP-1 expression. Conversely, SHP-1 has been found to be overexpressed in epithelial cancer, such as breast cancer (Tsui et al., 2006).

Next, SH2 domain-containing phosphatase 2 (SHP-2), encoded by the *PTPN11* gene, with a molecular weight of 68 kDa, is another member of the PTP family. SHP-2 shares a structure similar to SHP-1: both are composed of two SH2 domains at the N-terminus followed by a PTP domain and two tyrosine residues for phosphorylation by various stimuli at the C-terminus (Figure 1A). Comparable to SHP-1, SHP-2 is also auto-inhibited by its N-SH2 domain (Figure 1B). Activating SHP-2 mutations have been detected in many cancers, such as melanoma, acute myeloid leukemia (AML), lung cancer, colorectal cancer, etc. (Bentires-Alj et al., 2004; Miyamoto et al., 2008). This result suggests that *PTPN11* may be a proto-oncogene. Despite its oncogenic potential, SHP-2 plays a role in tissue development. This is illustrated as SHP-2 expression was found to be present in neurons during brain development. A loss-of-function mutation of SHP-2 in mice led to inhibition of sympathetic neurite outgrowth (Chen et al., 2002). Furthermore, SHP-2 deficiency is related to autoimmune disorders with varying defectives in organ development, such as Noonan syndrome and Leopard syndrome. *PTPN11* mutations were detected in 50 and 80% cases, respectively, in patients with these two syndromes (Table 1) (Shen et al., 2020).

**TABLE 1:** Hematopoietic and non-hematopoietic abnormalities *in vivo* mediated by dysregulated SHP-1, SHP-2, and SHIP.

	Hematopoietic	Non-hematopoietic
SHP-1	Impaired stemness of HSCs Macrophage autoimmune hyperactivation	Fatal pneumonitis
SHP-2	BM aplasia and lethality Defective HSCs in homing, self-renewal, and survival	Compromised sympathetic neurite outgrowth Hepatic inflammation and hepatocyte mortality Noonan syndrome and Leopard syndrome
SHIP	Impaired stemness of HSCs Increased mobilization of BM cells	Intestinal and lung inflammation

Lastly, SH2 domain-containing inositol phosphatase (SHIP) is a 145 kDa sized protein that is encoded by the *INPP5* (inositol polyphosphate-5-phosphatase) gene. SHIP interferes with the PI3K (phosphatidylinositol 3-kinase)/Akt pathway by dephosphorylating the P13K product PI(3,4,5)P3 to PI(3,4)P2 (Blunt and Ward, 2012). In the presence of SHIP, a controlled level of PI(3,4,5)P3 is produced, which activates downstream Akt and induces cell proliferation. Alternate transcriptional splice variants of SHIP encoding different isoforms of the protein have been characterized. One isoform of SHIP, SHIP-1, is expressed primarily in the hematopoietic system and suppresses the proliferation of hematopoietic progenitor cells (Fu et al., 2019). As shown in Figure 1A, the SH2 domain of SHIP is not involved in autoinhibition, but instead is responsible for membrane translocation and recruitment to upstream kinases. SHIP is activated by phosphorylation of Ser440 in the phosphatase domain by PKA (cyclic AMP-dependent protein kinase) (Zhang et al., 2009), or it is allosterically activated by binding of PI(3,4)P2 to the C2 domain (Ong et al., 2007). The dephosphorylation activity of SHIP to produce PI (3,4,5)P3 is carried out by both the Pleckstrin homology-related (PH-R) and phosphatase domain (Pauls and Marshall, 2017). In addition, the tyrosine residues Y915 and Y1022, located on the C-terminal region of SHIP, regulate the binding of the adapter protein and the activation of subsequent signaling (Lamkin et al., 1997). Furthermore, deficient SHIP led to inflammatory disorders *in vivo* since SHIP serves to suppress production of inflammatory cytokine by innate immune cells, such as neutrophils and basophils (Kerr, 2011). Severe inflammatory lung disease has been observed in SHIP knockout mice (Lo et al., 2019). Crohn’s disease-like intestinal inflammation and fibrosis were revealed in SHIP knockout mice and irradiated wild-type mice reconstituted with splenocytes from SHIP knockout mice (Kerr, 2011; Maxwell et al., 2011; Lo et al., 2019) as well (Table 1).

In this review, we will interpret SHP-1, SHP-2, and SHIP in terms of their association with cell surface receptors, downstream signaling pathways, and roles in hematopoiesis. We will also focus on how SHP-1, SHP-2, and SHIP are involved in hematopoietic malignant diseases, particularly in AML, and the current development of leukemic therapy involving manipulation of their expression.

## Cell Surface Receptors and Downstream Signaling Pathways

SH2 domain-containing phosphatase 1, SHP-2, and SHIP are recruited by and bound to multiple receptors on the cell surface through their SH2 domains. In myeloid cells, SHP-1 is associated with growth factor receptors, such as c-Kit (tyrosine protein kinase, CD117), and several immunoreceptor tyrosine-based inhibitory motif (ITIM) containing receptors, including paired immunoglobulin-like receptor B (PIR-B), leukocyte immunoglobulin-like receptor 1 (LIR-1), and leukocyte immunoglobulin-like receptor 2 (LIR-2; Zhang et al., 2000). In hematopoietic stem cells (HSCs), SHP-2 is activated by Kit, a mast/stem cell growth factor receptor (Kan et al., 2018). In addition, SHP-2 is expressed in stem cells of other tissues, whereas SHP-1 expression is mainly in hematopoietic cells. For example, in neural stem cells, SHP-2 is activated by fibroblast growth factor (FGF) receptor, Vascular endothelial growth factor (VEGF) receptor, and other receptor tyrosine kinases. SHP-2 is also present in mesenchymal stem/progenitor cells and satellite cells, where its activity is regulated by various tyrosine kinases on the cell membrane. SHIP, through its SH2 domain, binds to ITIM receptors such as FcγRIIB and killer cell immunoglobulin-like receptor (KIR) as well as immunoreceptor tyrosine-based activation motif (ITAM) receptors in hematopoietic cells (Dempke et al., 2018).

SH2 domain-containing phosphatase 1 negatively regulates hematopoietic cell proliferation through many intracellular signals. SHP-1 inhibits cytokine receptors, including Epo-R, IL3-R, and IL-2R, as well as growth factor receptors with intrinsic tyrosine kinase activity, such as CSF-1 and GM-CSF that reduce the proliferation of macrophages and granulocytes (Zhang et al., 2000). SHP-1 also suppresses the activation of the growth factor-induced signaling pathway of PI3K/Akt and nuclear factor-kappa B (NF-κB). SHP-1 regulates extracellular signal-related kinases (ERKs) and c-Jun-amino terminal kinases (JNKs) in a positive or negative manner. In addition, SHP-1 binds to erythropoietin (EPO) receptor and blocks subsequent activation of Janus kinase 2 (Jak2), resulting in downregulation of signal transducer and activator of transcription (STAT) (Chong and Maiese, 2007; Dempke et al., 2018). Most of the downstream factors of SHP-1 are also modulated by SHP-2, however, they are regulated in different ways. Upon induction by growth factor, SHP-2 promotes the PI3K/Akt pathway, ERK, and NF-κB through its association with signal regulatory protein α1 (SIRPα1) or Grb2-associated binder-1 (Gab1). Depending on the circumstance, SHP-2 can negatively or positively regulate STAT activation through Jak2 and JNK activation via Ras (Chong and Maiese, 2007; Dempke et al., 2018). Moreover, SHP-2 directly binds to and activates receptors for many growth factors and cytokines, such as IL-3 and GM-CSF (Tamir et al., 2000). Finally, SHIP dephosphorylates the product of its upstream kinase, P13K, and therefore has an inhibitory effect on downstream factors of P13K, including Akt, ERK, and NF-κB. SHIP also affects other kinases, including Burton tyrosine kinase (BTK) and phospholipase-C gamma (PLC-γ), and various transcription factors, such as nuclear factor of activated T cells (NFAT) (Blunt and Ward, 2012; Dempke et al., 2018).

## Function in Normal Hematopoiesis

Several receptor tyrosine kinases (RTK) have been identified in hematopoietic cells and are critical mediators of cell signaling (Reilly, 2003). RTKs respond to chemokines, other cytokines, and numerous ligands. Ligand-induced phosphorylation of the RTK tyrosine residues invokes physiological actions, including cell growth, differentiation, metabolism, migration, and survival (Ruvolo, 2019). Phosphatases serve to regulate the actions of RTK through dephosphorylation of tyrosine residues and are required for maintenance of hematopoiesis in the hematopoietic microenvironment. Aberrant tyrosine phosphorylation induced by an imbalance between the activity of RTKs and phosphatases, such as SHP-1, SHP-2, and SHIP, can lead to abnormal cell signaling and hematopoietic defects (Table 1).

SHP-1 and its isoforms are widely expressed in all hematopoietic lineages and maturation stages. SHP-1 inhibits RTK pathways activated by various growth factors and cytokines (Neel et al., 2003; Lorenz, 2009; Abram and Lowell, 2017). Therefore, SHP-1 negatively affects hematopoietic differentiation of embryonic stem cells (Paling and Welham, 2005). The expression of dominant-negative SHP-1 in embryonic stem cells increased the formation of myeloid colonies during differentiation and was reduced by the expression of wild-type SHP-1. Mice lacking SHP-1 exhibited a plethora of perturbations in their hematopoietic and immune systems. Defective myelopoiesis has been found in mice with the SHP-1 inactivation mutation. SHP-1 mutant mice demonstrated an enlarged neutrophil and monocyte population in peripheral blood and increased macrophage proliferation, contributing to the development of fatal pneumonitis (Table 1). In lymphocytes, SHP-1 stimulated cell growth and suppressed their oncogenic capacity (Tibaldi et al., 2011). Mice with specific deletion of SHP-1 in B cells or dendritic cells exhibited increased differentiation and autoimmunity of B-1a and Th1 cells (Pao et al., 2007; Kaneko et al., 2012). Loss of SHP-1 expression in tumor-specific T cells or natural killer cells promoted immune response and antitumor function in a mouse model of disseminated leukemia (Stromnes et al., 2012; Viant et al., 2014). Our previous *in vitro* study reported that HSCs from SHP-1 knockout mice have attenuated quiescence and impaired long-term self-renewal (Jiang et al., 2018). Therefore, we identified SHP-1 as a regulatory PTP to maintain the microenvironmental homeostasis of HSCs.

SH2 domain-containing phosphatase 2 is also required to maintain hematopoietic growth and homeostasis. SHP-2 contributes to cytokine-mediated signaling through several tyrosine kinases, and therefore loss of SHP-2 results in many dysfunctions in hematopoiesis (Nabinger and Chan, 2012; Rehman et al., 2019). The absence of the *PTPN11* gene in murine hematopoietic cells has been shown to result in BM (bone marrow) aplasia and lethality *in vivo* (Chan et al., 2011). Mice with *PTPN11* deletion also exhibited a rapid loss of functional HSCs and myeloid progenitors as well as reduced cellularity in the BM, spleen, and peripheral blood (Chan et al., 2011; Zhu et al., 2011). *PTPN11* knockout led to aberrant proliferation and promoted apoptosis of HSCs and progenitor cells *in vivo*, resulting in defects in homing, self-renewal, and survival (Zhu et al., 2011). Therefore, HSCs with deficient SHP-2 cannot reconstitute peripheral blood in lethally irradiated mouse recipients. SHP-2 is required not only for mouse hematopoiesis, but it is also necessary for human hematopoiesis. Upon SHP-2 knockout in human CD34^+^ cord blood cells, cell proliferation and colony formation decreased (Li et al., 2011). HSCs of a patient with a point mutation in *PTPN11*, eliminating the phosphatase activity of SHP-2 in human CD34^+^ cord blood cells, lost the ability to form colonies (Broxmeyer et al., 2013). These findings reveal a critical role for SHP-2 in the maintenance of functional HSCs and progenitors.

SH2 domain-containing inositol phosphatase is involved in the regulation of HSC proliferation and self-renewal. SHIP knockout induced an increase in the number of HSCs in the BM, spleen, and peripheral blood *in vivo* (Desponts et al., 2006). Mice with SHIP deletion demonstrated increased HSC cycling that was originally quiescent, which is justified by an increase in 5-FU sensitivity (Helgason et al., 2003). Although HSCs in SHIP knockout mice showed increased proliferation and reduced apoptosis, they also exhibited decreased homing ability to the BM after transplantation due to lower expression of CXCR4 and VCAM-1 receptors (Desponts et al., 2006). HSCs with SHIP knockout also revealed less self-renewal behavior *in vivo* as the expansion level of competitive repopulating cells (CRU) in the absence of SHIP was significantly lower than wild-type CRUs following transplantation (Helgason et al., 2003). Additionally, SHIP affects HSC adhesion in BM niches, and mobilization of primitive BM cells was expanded in SHIP knockout mice due to increased chemokine responsiveness (Helgason et al., 2003). These data demonstrate that SHIP plays a negative regulatory role in HSC proliferation and survival, and that SHIP is important in the maintenance of primitive hematopoietic cell homeostasis and regeneration.

## Relevance to Hematopoietic Disease and Leukemia

Dysregulation of SHP-1, SHP-2, and SHIP is associated with uncontrolled cell growth and metabolism, which results in the activation of multiple pro-oncogenic cascades and eventually leads to leukemia. In fact, mutations of these phosphatases are identified in a fair percentage of patients with leukemia, and abnormal levels of SHP-1, SHP-2, and SHIP have been detected in mouse models of leukemia (Dempke et al., 2018). This suggests that they are associated with leukemia development. Therefore, agents that modify the levels or activity of these phosphatases are currently investigated for potential leukemic therapies.

### SHP-1

Many studies have identified suppressive effects of SHP-1 on leukemia. For example, promotor methylation leading to silencing of SHP-1 has been reported in 10% of AML cases (Johan et al., 2005). Reexpression of SHP-1 by 5-Azacytidine, a DNA methyltransferase inhibitor, led to increased apoptosis of MV4-11 cells through down-regulation of STAT3 (Al-Jamal et al., 2015). In adult T cell leukemia/lymphoma, the SHP-1 protein has been shown to dephosphorylate and inactivate Sirtuin-1 (SIRT1) that repairs DNA of leukemia cells through homologous recombination (Yu et al., 2018). Upregulation of SHP-1 in Jurkat cells resulted in increased DNA damage, a higher incidence of apoptosis, and reduced colony formation *in vitro*. On the contrary, other studies indicate that the presence of SHP-1 is associated with the development of leukemia. *In vivo* myeloproliferative diseases induced by FMS like tyrosine kinase 3-internal tandem duplications (FLT3-ITD), a mutation present in approximately 30% of AML patients, were compromised by deletion of SHP-1 (Reich et al., 2020). Furthermore, our group has demonstrated that LAIR1-mediated SHP-1 activation recruited CAMK1 as an autonomous phosphatase signal adapter for downstream activation of CREB in AML cells, which contributes to the self-renewal of AML cells (Kang et al., 2015).

Likely due to the undetermined role of SHP-1 in the pathogenesis of AML, treatments that alter the expression level of SHP-1 do not currently exist for AML patient therapy. From 2009 to 2018, sodium stibogluconate (SS), a SHP inhibitor with a potent inhibitory effect on SHP-1, underwent several clinical trials and was proven to treat Leishmaniasis (Table 2). On the other hand, the antileukemia effect of SS was reported *in vitro* in early 2002 (Pathak et al., 2002). This study showed that NB4, a human AML cell line, exhibited higher differentiation, with cell growth arrest in S phase, and increased apoptosis following SS treatment. SS has also been shown to inhibit *in vitro* growth and induced differentiation of HL-60 and U937 cells. However, no study has confirmed a significant SS-mediated anti-AML effect *in vivo*, and this may be due to various bodily microenvironments weakening the efficacy of the drug. In addition to the lack of *in vivo* studies of the SHP-1 inhibitor, the clinical trial of AML treatments involving alterations of SHP-1 activity does not show a significant result. An AML preclinical trial consisting of a therapy that combines azacytidine and gemtuzumab ozogamicin (GO) aimed to enhance the cytotoxicity of the CD33 antibody against AML blasts through epigenetic modifications of *PTPN6* (Medeiros et al., 2018). Although 24% of participating patients obtained complete remission, an association between SHP-1 expression and clinical response was not found. The contradicting effects of SHP-1 in AML *in vitro* and the lack of significant findings *in vivo* indicate a lack of understanding of SHP-1 signaling pathways and mechanisms involving AML pathogenesis. This blind spot in knowledge must be explored more explicitly in order to better understand the therapeutic potential of targeting SHP-1 in AML.

**TABLE 2:** SHP and SHIP inhibitors with clinical trial or with tumor suppression effect *in vivo*.

	Name	IC50 or inhibition concentration	*In vivo* effect	Clinical trial time	Trial number/phase	Disease	Status
SHP-1 inhibitor	Sodium stibogluconate	Inhibits 99% of SHP-1 and SHP-2 activity at 10 and 100 μg/mL.	Growth inhibition of inoculated Renca tumors in BALB/c mice (Fan et al., 2005)	2007 2010 2018 2009	NCT00498979/Phase 1 NCT01067443/Phase 2 NCT03129646/Phase 3 NCT01661296/Phase 4	Stage IV Melanoma Primary Visceral Leishmaniasis Visceral Leishmaniasis Cutaneous Leishmaniasis	Completed in Jan 2012; negative response (Dempke et al., 2018) Completed in Jan 2012; positive response (Wasunna et al., 2016) Completed in Dec 2020; no result posted Completed in Dec 2011; no result posted
	TPI-1	40 nM	Growth inhibition of B16 melanoma tumors (Kundu et al., 2010)	No clinical trial			
SHP-2 inhibitor	TNO-155	0.011 μM	Growth inhibition of xenograft KYSE-520 tumor (LaMarche et al., 2020)	2021 2020 2020 2020	NCT04699188/Phase 1| Phase2 NCT04292119/Phase 1| Phase2 NCT04330664/Phase 1| Phase2 NCT04294160/Phase 1	Pulmonary and colorectal cancer Lung cancer and anaplastic Lymphoma Advanced cancer, metastatic cancer, malignant neoplastic disease Braf v600 colorectal cancer	Recruiting; estimated completion in Mar 2022 Recruiting; estimated completion in Mar 2023 Recruiting; estimated completion in Oct 2022 Recruiting; estimated completion in Aug 2023
	JAB-3068	N/A	N/A	2018	NCT03518554/Phase 1	Non-small cell lung cancer, head and neck cancer, esophageal cancer, other metastatic solid tumors	Recruiting; estimated completion in Jul 2021
	RMC-4630	N/A	N/A	2018 2019	NCT03634982/Phase 1 NCT03989115/Phase 1| Phase 2	Relapsed/refractory solid tumor Relapsed/refractory solid tumor	Recruiting; estimated completion in Oct 2021 Recruiting; estimated completion in Apr 2022
	RLY-1971	N/A	N/A	2020	NCT04252339/Phase 1	Advanced or metastatic solid tumors	Recruiting; estimated completion in Apr 2022
	IACS-13909	15.7 nM	Growth suppression of xenograft KYSE-520 tumor and suppression of MV4-11 induced FLT3-ITD AML (Sun et al., 2020)	No clinical trial			
	SHP 394	23 nM	Reduced tumor volume and raised tumor regression in mice carrying Detroit-562 pharyngeal carcinoma cells (Sarver et al., 2019)	No clinical trial			
	SHP099	70 nM	Reduced tumor volume of KYSE520 xenografts, reduced number of circulating leukemia cells and reduced splenomegaly in mice with patient-derived FLT3-ITD AML (Chen et al., 2016)	No clinical trial			
SHIP inhibitor	3α-Aminocholestane	2.5 μM	Reduced multiple myeloma (MM) growth, reduced number of circulating cancer cells, and enhanced survival rate (Fuhler et al., 2012)	No clinical trial			

SH2 domain-containing phosphatase 1 has also demonstrated oncogenic properties in other leukemias. From the *in vitro* assay of chronic lymphocytic leukemia (CLL), SHP-1 underwent differential phosphorylation and, resultantly, exhibited differential functions and cellular localizations (Tibaldi et al., 2017). SHP-1 with phospho-S591 supported aberrant Lyn-dependent tyrosine phosphorylation of proteins in the cytosol of CLL cells and eventually formed a network of anti-apoptotic signaling. In B cell acute lymphoblastic leukemia (B-ALL), inducible ablation of SHP-1 reduced proliferation and stemness and increased cell cycle arrest in murine B-ALL cells. *In vivo* deletion of SHP-1 also extended the latency of leukemia and improved the survival rate of mice (Chen Z. et al., 2015). In patients with acute leukemia (AL), the differential expression of SHP-1 and the six-cytokine signaling suppressor (SOCS6) has been detected. SHP-1 and SOCS6 mRNA levels tended to be higher among patients in AL remission than in newly diagnosed patients. Therefore, the expression of SHP-1 and SOCS6 is associated with favorable outcomes, suggesting an anticancer property in AL and potential targets for gene therapy (Liu et al., 2017).

A SHP-1 inhibitor specific for leukemia treatment has not yet been developed. SS, a SHP-1 antagonist that showed effective suppression on inoculated tumors *in vivo*, underwent two Phase I clinical trials for malignant melanoma (Table 2). However, the outcome was poor, demonstrated by no objective response, life-threatening events in 68% of patients, and side effects such as pancreatitis, BM suppression, and nausea (Dempke et al., 2018). The pessimistic results of these clinical trials further imply an elusive role of SHP-1 in tumor development, and additional is needed to elucidate the potential of SHP-1 as a drug target for tumor treatment.

### SHP-2

SH2 domain-containing phosphatase 2 has been determined to have an oncogenic effect on cell proliferation and growth. Mutations in the N-SH2 domain of SHP-2 cause constitutive activation of the SHP-2 protein in the hematopoietic stem and progenitor compartment, resulting in the development of clinical leukemia. A *PTPN11* mutation was common in diagnosed patients with secondary AML (Makishima et al., 2017), relapsed pediatric AML (Farrar et al., 2016), and acute lymphoblastic leukemia (ALL; Oshima et al., 2016). The activating mutation of SHP-2 has been identified in 10% of AML cases (Dempke et al., 2018). The prevalence of *PTPN11* mutations has been found to be higher in patients over 60 years of age and associated with a poor prognosis (Tsai et al., 2016). Secondary AML patients with *PTPN11* mutations tended to progress rapidly and have lower overall survival (Makishima et al., 2017). The *PTPN11* mutation exists not only in older patients with AML but also in children with *de novo* AML. A study identified the substitution of trinucleotides at position 211–213 in 24 pediatric patients as well as other *PTPN11* mutations in children with Juvenile myelomonocytic leukemia (JMML) and myelodysplastic syndrome (MDS; Tartaglia et al., 2003). Interestingly, almost all of the mutations identified in these leukemias are located in the N-SH2 domain, which confirms the essential role of the N-SH2 domain in regulating SHP-2 activity and thus the growth and proliferation of hematopoietic cells. The SHP-2 protein also plays an oncogenic role in the development of AML from a molecular perspective. SHP-2 inhibition negatively regulates the activation of downstream factors in FLT3-ITD pathways, which was illustrated *in vitro* as SHP-2 inhibition led to proliferation of blast cells with FLT3-ITD. Mutation of the FLT3-ITD residue that recruits SHP-2 also reduced FLT3-ITD-induced myeloproliferative disease *in vivo* (Richine et al., 2016). Furthermore, SHP-2 is involved in the signaling pathway of leukocyte immunoglobulin-like receptor B4 (LILRB4), an immunoreceptor tyrosine-based inhibition motif-containing receptor (Deng et al., 2018). LILRB4 inhibited T cell proliferation and improved the infiltration of AML cells into organs both *in vitro* and *in vivo*, and these effects were reversed by knockout of SHP-2 in human AML cell lines.

In addition, the mixed-lineage leukemia (MLL) translocation, *MLL-AF10*, is known to occur in patients with a *G503A* mutation in *PTPN11*. With the inclusion of both the *MLL-AF10* and *G503A* mutations in a mouse model, there was an accelerated rate of disease development compared to the control group (Fu et al., 2017). *PTPN11 E76K* mutation resulted in increased proliferation of mouse HSCs *in vitro*. Transplantation of HSCs co-expressing *PTPN11 E76K* and *MLL-AF9* fusion oncogenes induced a lower survival rate and more severe AML phenotype *in vivo*, such as splenomegaly (Chen L. et al., 2015). Given that SHP-2 imposes an oncogenic effect on hematopoietic cells and that its mutation has been identified in multiple malignancies, efforts have been devoted to discovering inhibitors of SHP-2 and examining their effects against cancers. For example, IACS-13909 (Sun et al., 2020) and SHP099 (Chen et al., 2016) inhibited the proliferation of human AML cell lines and reduced tumor burden derived from xenograft AML cells *in vivo* (Table 2). Importantly, there are several SHP-2 inhibitors, including TNO-155 (LaMarche et al., 2020; Liu et al., 2021), currently in clinical trials for multiple solid tumors and cancers that will be completed in spring 2022. Although results have yet to be announced, the potential of SHP-2 inhibitors as a treatment option for AML is expected given the strong oncogenicity of SHP-2 and its entanglement in AML oncogenic pathways.

### SHIP

SH2 domain-containing inositol phosphatase 1, a SHIP isoform, has been proposed to play a regulatory role in the development of AML. This is because approximately 50–70% of patients with AML demonstrated constitutive activation of the SHIP-1-regulated P13K/Akt pathway, and approximately 3% displayed a missense mutation in *INPP5D* (Täger et al., 2017). A higher level of SHIP-1 correlated with prolonged overall survival among 290 patients with AML as well. Furthermore, overexpression of SHIP-1 reduced the proliferation of CD34^+^ cells in AML patients (Metzner et al., 2009). Studies based on AML cells and the AML mouse model also supported that SHIP-1 acts as an AML suppressor. SHIP-1 transfection to THP-1, a human AML cell line lacking endogenous expression of SHIP-1, resulted in a higher proportion of apoptotic cells. Moreover, SHIP-1 was significantly down-regulated in patients with late-stage MDS, and SHIP-1 expression reduced the number of colonies formed by primary patient myeloid leukemia blasts (Lee et al., 2012). Furthermore, SHIP-1 overexpression extended the life span of the NSG mice model transplanted with human AML cells (Täger et al., 2017).

Considering the role of SHIP-1 as an AML suppressor, factors that suppress SHIP-1 have been investigated. SHIP-1 has been demonstrated to be targeted and down-regulated by miR-155. Compared to normal CD34^+^ cells, miR-155 expression was significantly higher in cells isolated from patients with late-stage MDS (Lee et al., 2012). Therefore, it is suggested that inhibition of miR-155 can restore suppression of AML by SHIP-1. One study identified Silvestrol as a miR-155 inhibitor and found it to down-regulate miR-155 levels in MV4-11 cells as well as inhibit the growth of MV4-11 and THP-1 cells (Brooks et al., 2010). Silvestrol treatment also improved the survival rate of AML mice *in vivo* and increased *in vitro* apoptosis of primary blasts from AML patients. Another study showed that MLN4924 decreased the miR-155 level in AML cells (Khalife et al., 2015). Treatment of MV4-11 cells with MLN4924 resulted in a reduced level of miR-155, upregulation of SHIP-1, suppression of the P13K/Akt pathway, and monocytic differentiation. MLN4924 also reduced the viability of MV4-11 cells and blasts from AML patients. Furthermore, the administration of MLN4924 to an AML mouse model prolonged the survival period, although all treated mice eventually died.

Based on these studies, SHIP-1 is recognized as an AML suppressor via attenuation of the P13K/Akt pathway and reduction of subsequent cell proliferation. However, some studies exhibit dual roles of SHIP-1. In some cases, SHIP-1 amplified survival or proliferative signals in neoplastic cells. The enzyme product of SHIP-1, PI(3,4) P2, has been shown to have higher affinity for Akt and led to more potent activation of the Akt pathway *in vitro* (Brooks et al., 2010). Treatment of the KG-1 AML cell line with 3α-aminocholestane (3-AC), a SHIP-1 inhibitor, resulted in reduced cell viability *in vitro*. Furthermore, SHIP-1 expression in patients with AML are largely variable and are not inversely associated with activated Akt level. Therefore, the role of SHIP-1 in the P13K/Akt pathway and AML cell proliferation needs to be clarified further. Considering that the antileukemic effect of 3-AC has not been tested in an AML mouse model, effects of SHIP-1 inhibition *in vivo* may provide more insight regarding the role and function of the phosphatase in AML.

## Concluding Remarks and Perspectives

The identification of SHP-1, SHP-2, and SHIP and their downstream signaling in hematopoietic cells provides new clues to the development and treatment of leukemia. The studies reviewed here demonstrate that an abnormal level of these phosphatases, instead of altered signaling pathways, is associated with aberrant proliferation of hematopoietic cells and leukemia development. Abnormal expression levels derive either from genetic mutations, such as a loss-of-function mutation of SHP-1 or a gain-of-function SHP-2 mutation, or dysregulated regulatory signals, such as upregulated miR-155 levels that inhibit transcription of the SHIP-1 gene in AML patients. Therefore, SHP-1, SHP-2, and SHIP can be potential targets for anti-leukemia therapy. However, scientists are still not in uniform agreement on the true role of these phosphatases in leukemia, which imposes challenges on the development of leukemia-treating reagents that manipulate expression levels of these proteins. Until now, most studies have supported the oncogenic property of SHP-2 and the tumor suppressor property of SHIP, but the effects of SHP-1 on leukemia are still controversial. Consequently, future studies must focus on confirming the effects of SHP-1 and its downstream pathways in different types of leukemia. In regard to SHP-2, it is necessary to refine its antagonists to suppress leukemia development *in vivo* for clinical trials. Furthermore, SHP-2 inhibitors, which have already undergone clinical trials to treat other tumors and cancers, need to be evaluated for their efficacy in the treatment of leukemia. Finally, miR-155 inhibitors, which upregulate SHIP-1, exhibit suppressive effects in AML *in vivo* and should also be refined for AML clinical trials.

## Author Contributions

FH and CW wrote the manuscript and prepared figures. CS provided critical comments and proofreading the manuscript. MC provided comments and joined the discussion. XK organized the structure of the manuscript, provided critical comments and proofreading the manuscript.

## Conflict of Interest

The authors declare that the research was conducted in the absence of any commercial or financial relationships that could be construed as a potential conflict of interest.

## Publisher’s Note

All claims expressed in this article are solely those of the authors and do not necessarily represent those of their affiliated organizations, or those of the publisher, the editors and the reviewers. Any product that may be evaluated in this article, or claim that may be made by its manufacturer, is not guaranteed or endorsed by the publisher.
